# Supplementary material for: High frequency body site translocation of nosocomial Pseudomonas aeruginosa
Source: Nat Commun. 2025 Nov 25;16:9862. doi: 10.1038/s41467-025-66088-x (PMC12647771; doi:10.1038/s41467-025-66088-x)
Supplement: Supplementary file 1 — Supplementary information [file 41467_2025_66088_MOESM1_ESM.pdf]

High frequency body site translocation of nosocomial *Pseudomonas aeruginosa*

SUPPLEMENTARY INFORMATION

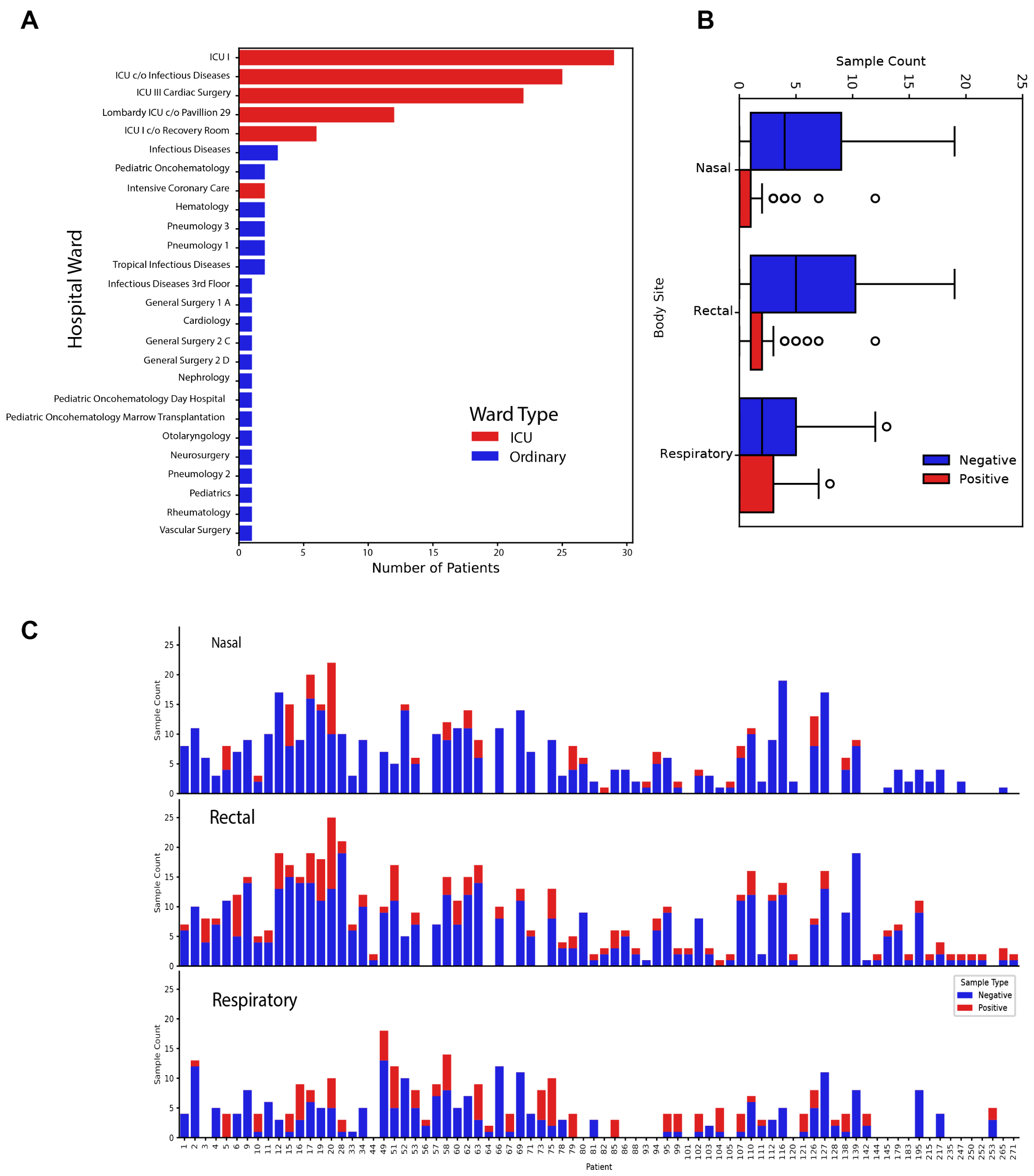

Supplementary Fig.1: Patients with *P. aeruginosa* were mainly found within the ICU wards of the hospital

A, The number of patients residing in each ward coloured by ward type. Intensive care units (ICU) in red, Ordinary wards in blue. B, The boxplot shows the numbers of positive (red) and negative (blue) samples for patients at each body site. Outliers are indicated by black, white

fileld circles. C, The number of positive (red) and negative (blue) samples per patient within each body site. Empty columns show where a patient wasn't sampled at that site.

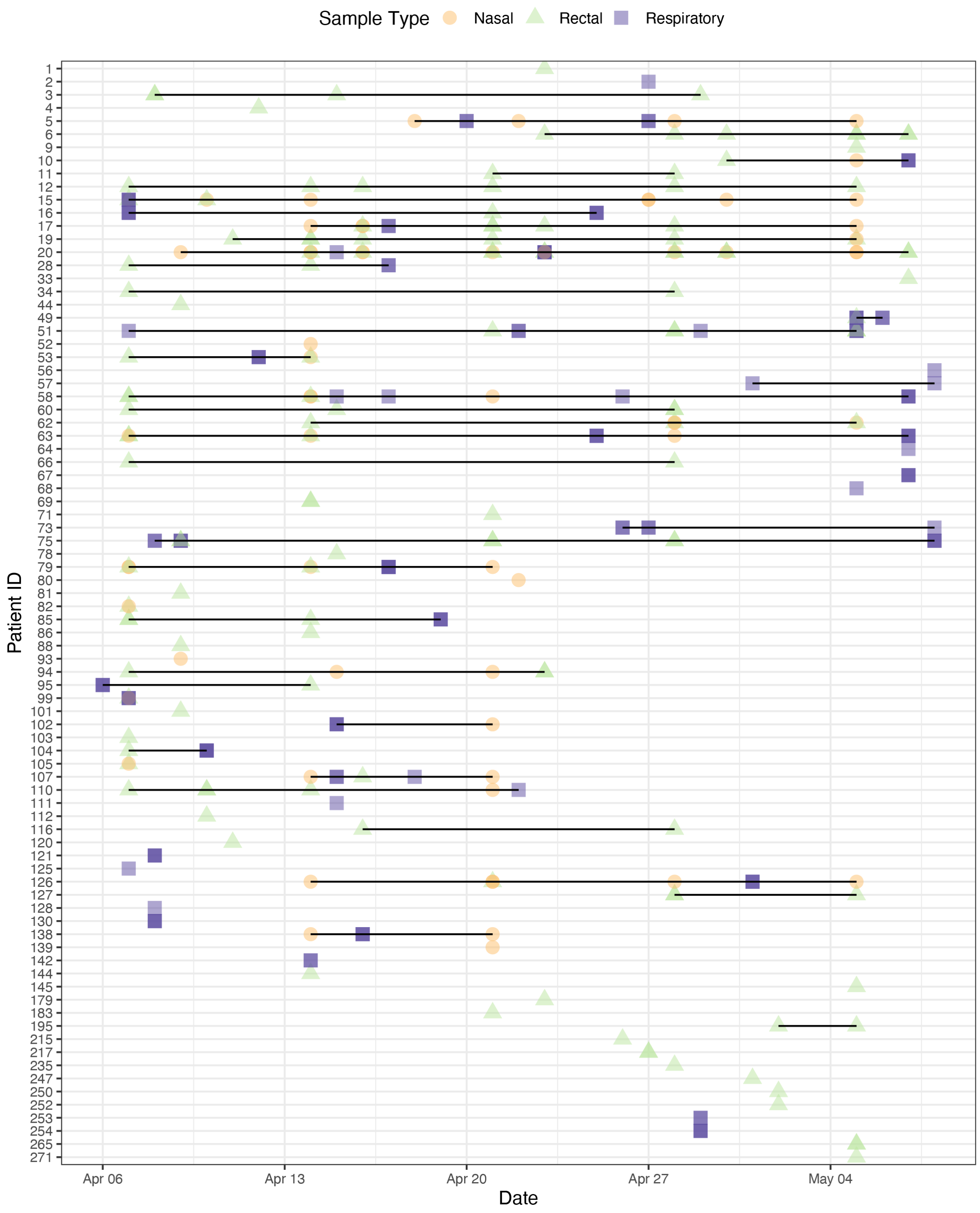

Supplementary Fig.2: Sampling of patients over the one-month study period.

P. aeruginosa positive samples were coloured by sample type (green = rectal, purple = respiratory, yellow = nasal). The black lines indicate where there were more than one positive samples at separate timepoints for each patient.

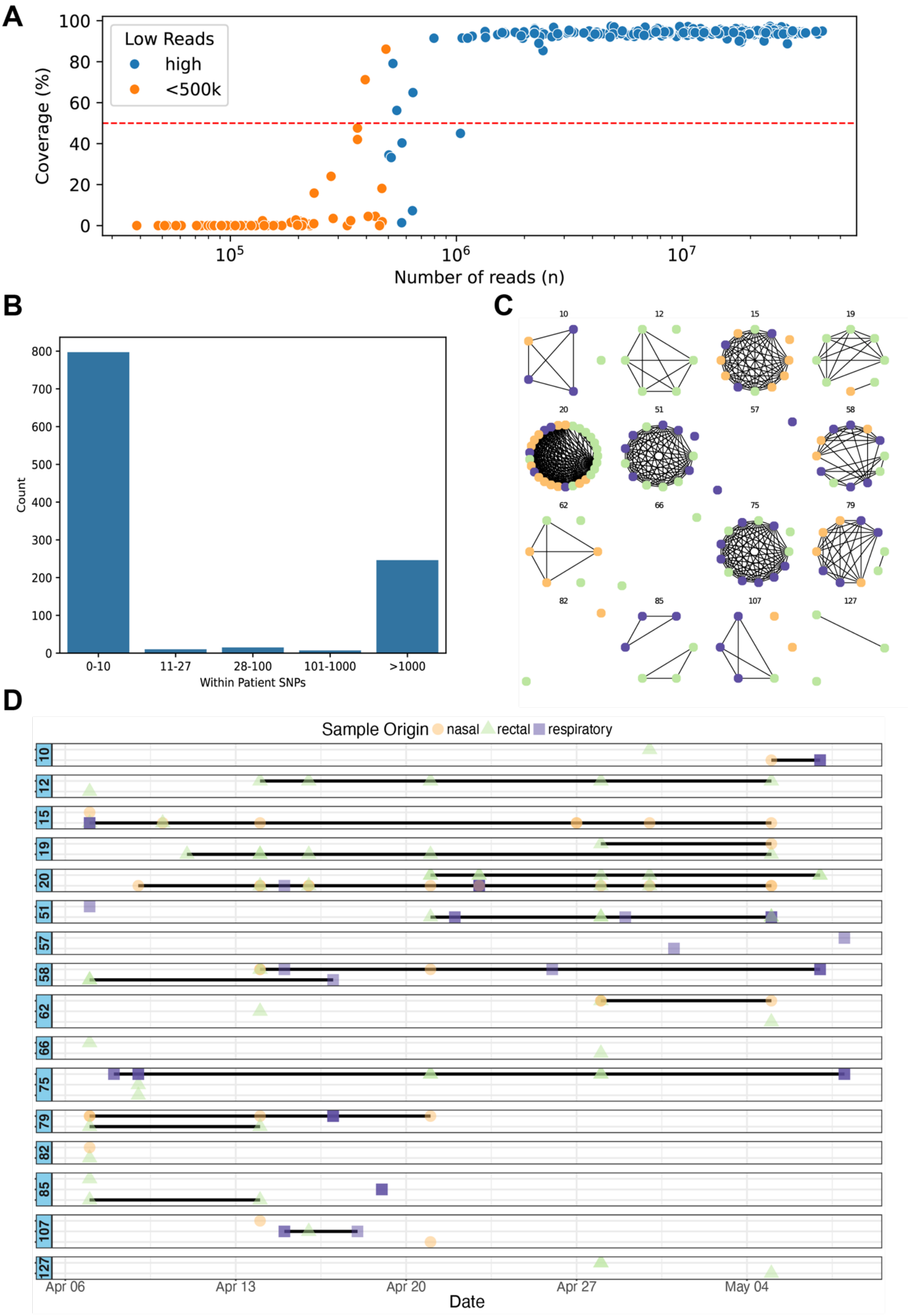
A
Low Reads
high
<500k
Coverage (%)
Number of reads (n)
B
Count
0-10
11-27
28-100
101-1000
>1000
Within Patient SNPs
C
10
12
15
19
20
51
57
58
62
66
75
79
82
85
107
127
D
Sample Origin
nasal
rectal
respiratory
Apr 06
Apr 13
Apr 20
Apr 27
May 04
Date

Supplementary Fig.3: Sixteen patients with multiple clones.

A, Points represent each whole genome sequencing sample with the number of reads on the x axis and percentage coverage on the y axis. Points coloured in orange represent samples with less than 500,000 *P. aeruginosa* reads, point in blue represent anything greater than 500,000. B, Within patient SNPs at five defined thresholds, 0-10, 0-27 (Weimann *et al.,* 2024), 28-100, and 101-1000, and anything 1001 and greater. C, Network graphs are labelled with patient number. Each node represents a sample (green = rectal, purple = respiratory, yellow = nasal) and each edge shows a difference of less than 100 SNP differences between samples. No edges show that the samples were greater than 100 SNPs apart and would constitute a separate clone. D, Each point indicates a sample positive for *P. aeruginosa* coloured by sample type (green = rectal, purple = respiratory, yellow = nasal). Where clones persisted in separate time point, a black line is used to join the points.

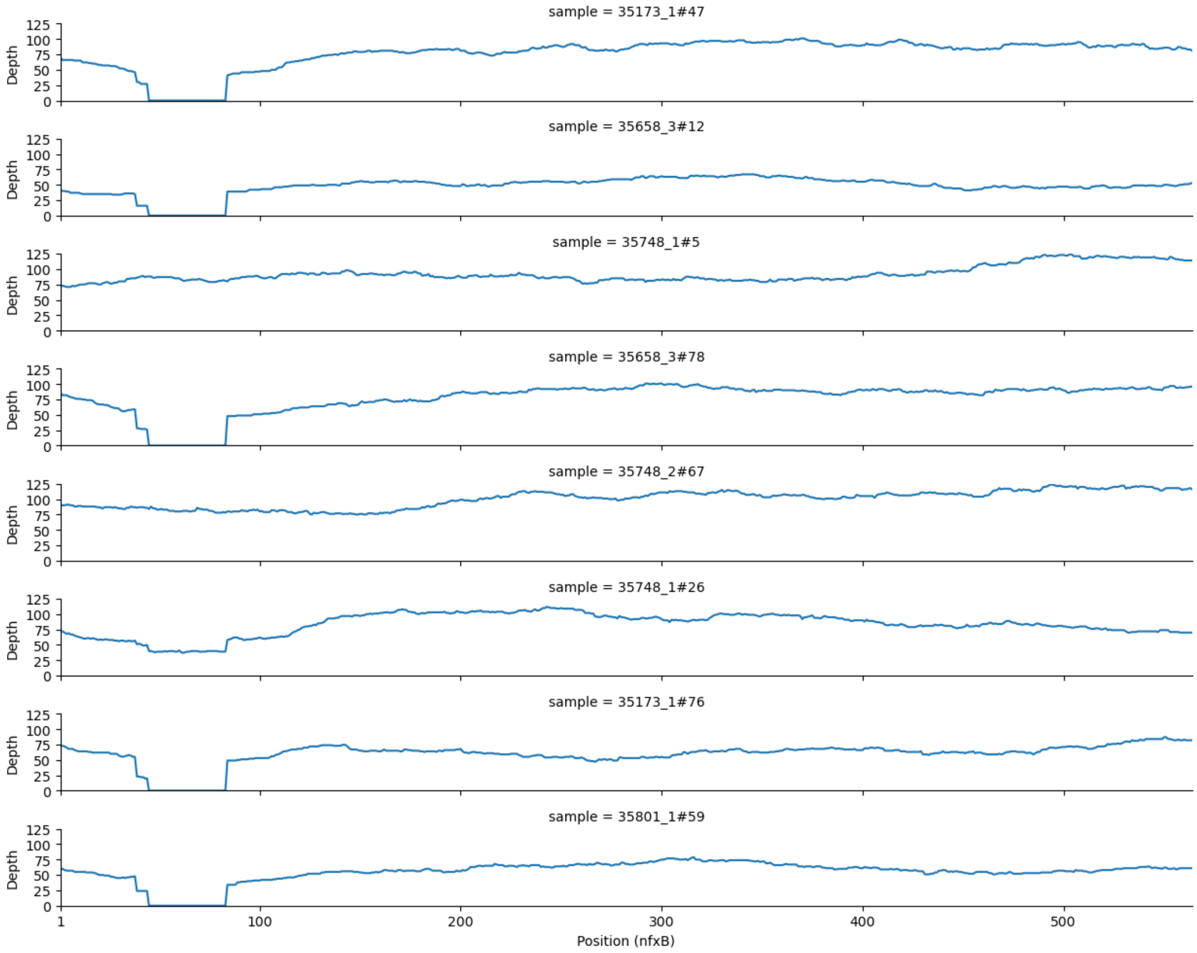

Supplementary Fig.4: Missing coverage in *nfxB* genes within patient 20 clone B.

The depth at each position in the *nfxB* gene was calculated using Samtools. The plot shows the coverage across the *nfxB* gene and reveals additional mutations (large deletions) within the sequence.
