# Supplementary material for: High frequency body site translocation of nosocomial Pseudomonas aeruginosa
Source: Nat Commun. 2025 Nov 25;16:9862. doi: 10.1038/s41467-025-66088-x (PMC12647771; doi:10.1038/s41467-025-66088-x)
Supplement: Supplementary file 2 — Description of Additional Supplementary Files [file 41467_2025_66088_MOESM2_ESM.pdf]

Description of Additional Supplementary Files

Supplementary Data 1: Dataset provided in excel file. Organism isolation data from blood cultures for patients within the study.

Supplementary Data 2: Dataset provided in excel file. Organism isolation data from bronchoalveolar lavage (BAL) cultures for patients within the study.

Supplementary Data 3: Dataset provided in excel file. P. aeruginosa sample metadata

Included within the table are ENA accession numbers, sample types, sequence type, and fastbaps clusters.
