# Supplementary material for: High frequency body site translocation of nosocomial Pseudomonas aeruginosa
Source: Nat Commun. 2025 Nov 25;16:9862. doi: 10.1038/s41467-025-66088-x (PMC12647771; doi:10.1038/s41467-025-66088-x)
Supplement: Supplementary file 6 — Reporting summary [file 41467_2025_66088_MOESM6_ESM.pdf]

nature portfolio

Corresponding author(s): Dr Josephine M Bryant
Last updated by author(s): Sep 29, 2025

# Reporting Summary

Nature Portfolio wishes to improve the reproducibility of the work that we publish. This form provides structure for consistency and transparency in reporting. For further information on Nature Portfolio policies, see our Editorial Policies and the Editorial Policy Checklist.

## Statistics

For all statistical analyses, confirm that the following items are present in the figure legend, table legend, main text, or Methods section.

| n/a | Confirmed | |
|---|---|---|
| ☐ | ☒ | The exact sample size (*n*) for each experimental group/condition, given as a discrete number and unit of measurement |
| ☐ | ☒ | A statement on whether measurements were taken from distinct samples or whether the same sample was measured repeatedly |
| ☐ | ☒ | The statistical test(s) used AND whether they are one- or two-sided<br>*Only common tests should be described solely by name; describe more complex techniques in the Methods section.* |
| ☒ | ☐ | A description of all covariates tested |
| ☐ | ☒ | A description of any assumptions or corrections, such as tests of normality and adjustment for multiple comparisons |
| ☐ | ☒ | A full description of the statistical parameters including central tendency (e.g. means) or other basic estimates (e.g. regression coefficient) AND variation (e.g. standard deviation) or associated estimates of uncertainty (e.g. confidence intervals) |
| ☐ | ☒ | For null hypothesis testing, the test statistic (e.g. *F*, *t*, *r*) with confidence intervals, effect sizes, degrees of freedom and *P* value noted<br>*Give P values as exact values whenever suitable.* |
| ☒ | ☐ | For Bayesian analysis, information on the choice of priors and Markov chain Monte Carlo settings |
| ☒ | ☐ | For hierarchical and complex designs, identification of the appropriate level for tests and full reporting of outcomes |
| ☒ | ☐ | Estimates of effect sizes (e.g. Cohen's *d*, Pearson's *r*), indicating how they were calculated |

*Our web collection on statistics for biologists contains articles on many of the points above.*

## Software and code

Policy information about availability of computer code

Data collection

ncbi-datasets-cli v15.9.1
get-assemblies v0.10.0

Data analysis

Code used in this study has been deposited: https://www.ebi.ac.uk/ena/browser/view/PRJEB39567

Additional tools used:

Python v3.1
Pysam v0.22.1
Biopython v1.84
Graphviz v12.0.0
Matplotlib v3.9.2
Matplotlib-venn v1.1.1
Networkx v3.4.2
Numpy v2.1.2
Pandas v 2.2.3
Scipy v1.14.1
Seaborn v0.13.2
Seqtk v1.3-r106
Bactgen scripts https://github.com/sanger-pathogens/bact-gen-scripts (v0.3)
Snp-sites v2.5.1


April 2023

Gubbins v3.3.5
Raxml-ng v1.1.1
Samtools v1.21
Snp-dists v0.7.0
MUSCLE v3.8.1551
R v4.4.3
Phytools v2.3-0
Tidyverse v2.0.0
Ape v5.8
ggtree v3.12.0
ggplot2 v3.5.1
dplyr v1.1.4
phangorn v2.12.1
doParallel v1.0.17
lubridate v1.9.3
foreach v1.5.2
stringr v1.5.1
viridis v0.6.5
reshape2 v1.4.4

For manuscripts utilizing custom algorithms or software that are central to the research but not yet described in published literature, software must be made available to editors and reviewers. We strongly encourage code deposition in a community repository (e.g. GitHub). See the Nature Portfolio guidelines for submitting code & software for further information.

## Data

Policy information about availability of data

All manuscripts must include a data availability statement. This statement should provide the following information, where applicable:

- Accession codes, unique identifiers, or web links for publicly available datasets
- A description of any restrictions on data availability
- For clinical datasets or third party data, please ensure that the statement adheres to our policy

Genome sequencing was deposited to ENA with accession numbers available in Extended Data Table.1.
ERS5293974, ERS5348395, ERS5347534, ERS5347566, ERS5294225, ERS5347679, ERS5294354, ERS5294098, ERS5286735, ERS5294018, ERS5286938, ERS5346999, ERS5287053, ERS5286839, ERS5294258, ERS5294015, ERS5347445, ERS5347320, ERS5346995, ERS5286631, ERS5286787, ERS5348425, ERS5286712, ERS5347526, ERS5287292, ERS5294008, ERS5287067, ERS5286467, ERS5294252, ERS5287202, ERS5287347, ERS5078686, ERS5287390, ERS5286653, ERS5294058, ERS5347420, ERS5294380, ERS5286630, ERS5286388, ERS5348487, ERS5347612, ERS5286782, ERS5348365, ERS5294196, ERS5293933, ERS5347432, ERS5294009, ERS5294254, ERS5347564, ERS5347677, ERS5348456, ERS5347524, ERS5294123, ERS5294386, ERS5286912, ERS5347554, ERS5347674, ERS5348489, ERS5294059, ERS5346420, ERS5347387, ERS5294315, ERS5347668, ERS5347632, ERS5286754, ERS5347545, ERS5346500, ERS5294042, ERS5286603, ERS5287132, ERS5213377, ERS5293889, ERS5078699, ERS5213293, ERS5078735, ERS5287352, ERS5293877, ERS5347547, ERS5347648, ERS5287209, ERS5346598, ERS5287148, ERS5294362, ERS5287304, ERS5294363, ERS5348480, ERS5293835, ERS5348481, ERS5294253, ERS5294107, ERS5346439, ERS5347572, ERS5287069, ERS5348368, ERS5286724, ERS5287466, ERS5347041, ERS5374384, ERS5287070, ERS5348458, ERS5347412, ERS5286481, ERS5287201, ERS5294194, ERS5294061, ERS5286667, ERS5286417, ERS5286844, ERS5374258, ERS5286816, ERS5287153, ERS5294318, ERS5287010, ERS5286963, ERS5287394, ERS5287312, ERS5294207, ERS5293948, ERS5346949, ERS5347622, ERS5294265, ERS5348375, ERS5294022, ERS5348433, ERS5347616, ERS5293950, ERS5287065, ERS5374333, ERS5286956, ERS5348377, ERS5294208, ERS5347477, ERS5287008, ERS5347641, ERS5294340, ERS5286659, ERS5287027, ERS5286897, ERS5347522, ERS5287247, ERS5374240, ERS5294086, ERS5348469, ERS5286824, ERS5294216, ERS5348455, ERS5287060, ERS5294135, ERS5294409, ERS5348476, ERS5348482, ERS5294365, ERS5294346, ERS5287274, ERS5214241, ERS5294045, ERS5287382, ERS5294299, ERS5293853, ERS5213280, ERS5346449, ERS5286565, ERS5374322, ERS5347688, ERS5286942, ERS5287446, ERS5293972, ERS5287043, ERS5348394, ERS5287139, ERS5286814, ERS5294222, ERS5294257, ERS5348424, ERS5286767, ERS5294013, ERS5347602, ERS5286795, ERS5286699, ERS5287340, ERS5294248, ERS5294004, ERS5286740, ERS5348419, ERS5347624, ERS5347504, ERS5294411, ERS5347382, ERS5294138, ERS5213306, ERS5348371, ERS5287242, ERS5287404, ERS5294203, ERS5348372, ERS5346684, ERS5293942, ERS5294204, ERS5347591, ERS5348498, ERS5294255, ERS5294266, ERS5287199, ERS5348420, ERS5348472, ERS5293854, ERS5294090, ERS5294413, ERS5294100, ERS5294357, ERS5294063, ERS5348497, ERS5286643, ERS5294320, ERS5347314, ERS5287130, ERS5346565, ERS5347681, ERS5347571, ERS5293820, ERS5287145, ERS5294387, ERS5287314, ERS5294355, ERS5348491, ERS5294127, ERS5287336, ERS5294393, ERS5347552, ERS5213317, ERS5287412, ERS5287167, ERS5287453, ERS5347584, ERS5293860, ERS5287287, ERS5286556, ERS5293845, ERS5287342, ERS5347582, ERS5293858, ERS5286598, ERS5293887, ERS5286570, ERS5293806, ERS5287384, ERS5293886, ERS5293952, ERS5348364, ERS5293932, ERS5287366, ERS5294195, ERS5348383, ERS5078747, ERS5293956, ERS5294212, ERS5347614, ERS5347594, ERS5287310, ERS5347656, ERS5293996, ERS5294240, ERS5287439, ERS5346519, ERS5287460, ERS5293865, ERS5287294, ERS5287141, ERS5287409, ERS5287206, ERS5287403, ERS5287197, ERS5293824, ERS5213302, ERS5347615, ERS5293928, ERS5347410, ERS5287108, ERS5293815, ERS5293895, ERS5287179, ERS5293829, ERS5293834, ERS5294202, ERS5287125, ERS5287344, ERS5286752, ERS5287124, ERS5287424, ERS5287340, ERS5287420, ERS5347547, ERS5293808, ERS5347560, ERS5347685, ERS5347620, ERS5347634, ERS5347578, ERS5294342, ERS5294088, ERS5347562, ERS5287458, ERS5286836, ERS5348492, ERS5287170, ERS5347556, ERS5346432, ERS5287426, ERS5294238, ERS5293873, ERS5293842, ERS5294056, ERS5348386, ERS5294313, ERS5287305, ERS5293811, ERS5294120, ERS5293960, ERS5347682, ERS5294110

## Research involving human participants, their data, or biological material

Policy information about studies with human participants or human data. See also policy information about sex, gender (identity/presentation), and sexual orientation and race, ethnicity and racism.

| | |
|---|---|
| Reporting on sex and gender | Not relevant |
| Reporting on race, ethnicity, or other socially relevant groupings | Not relevant |

| | |
|---|---|
| Population characteristics | Not relevant |
| Recruitment | Not relevant |
| Ethics oversight | Not relevant |

Note that full information on the approval of the study protocol must also be provided in the manuscript.

# Field-specific reporting

Please select the one below that is the best fit for your research. If you are not sure, read the appropriate sections before making your selection.

☒ Life sciences ☐ Behavioural & social sciences ☐ Ecological, evolutionary & environmental sciences

For a reference copy of the document with all sections, see nature.com/documents/nr-reporting-summary-flat.pdf

# Life sciences study design

All studies must disclose on these points even when the disclosure is negative.

| | |
|---|---|
| Sample size | There were 385 sets of deconvoluted Pseudomonas aeruginosa reads that were used for bioinformatic analysis. Sample size calculations were not relevant as this was an observational exploratory study without a prior hypothesis. This is the largest sample size generated for this kind of study generated thus far. |
| Data exclusions | No data were excluded. |
| Replication | Genome sequencing data are available in publicly accessible repositories and code for bespoke analysis is available on GitHub (https://github.com/Lewis-W-S-Fisher/cocov_paper). |
| Randomization | No experiments were performed as part of this study. Randomization was performed as part of the simulation experiments, which we repeated 10,000 times to ensure any individual patient/ward biases were controlled for. The most important covariate in this study was patient ID which was included in all phylogenetic analyses and statistical tests where only one representative sample or average value was considered per patient. |
| Blinding | The work did not require group allocation or blinding as this was an exploratory study without an initial hypothesis being tested. |

# Reporting for specific materials, systems and methods

We require information from authors about some types of materials, experimental systems and methods used in many studies. Here, indicate whether each material, system or method listed is relevant to your study. If you are not sure if a list item applies to your research, read the appropriate section before selecting a response.

## Materials & experimental systems

| n/a | Involved in the study | |
|---|---|---|
| ☒ | ☐ | Antibodies |
| ☒ | ☐ | Eukaryotic cell lines |
| ☒ | ☐ | Palaeontology and archaeology |
| ☒ | ☐ | Animals and other organisms |
| ☒ | ☐ | Clinical data |
| ☒ | ☐ | Dual use research of concern |
| ☒ | ☐ | Plants |

## Methods

| n/a | Involved in the study | |
|---|---|---|
| ☒ | ☐ | ChIP-seq |
| ☒ | ☐ | Flow cytometry |
| ☒ | ☐ | MRI-based neuroimaging |

## Plants

| | |
|---|---|
| Seed stocks | Not relevant |
| Novel plant genotypes | Not relevant |
| Authentication | Not relevant |
